# The continuum of care for dementia: needs, resources and practice in China

**DOI:** 10.7189/jogh.09.020321

**Published:** 2019-12

**Authors:** Huali Wang, Hengge Xie, Qiumin Qu, Wei Chen, Yongan Sun, Nan Zhang, Yu Liu, Tao Li, Kit Yee Chan, Serge Gauthier, Xin Yu, on behalf of the Global Dementia Prevention Program (GloDePP)

**Affiliations:** 1 Dementia Care and Research Center, Peking University Institute of Mental Health (Sixth Hospital), Beijing, China; 2 Beijing Dementia Key Lab, NHC Key Laboratory of Mental Health, Beijing, China; 3 National Clinical Research Center for Mental Disorders, Beijing, China; 4 Department of Geriatric Neurology, Chinese PLA General Hospital, Beijing, China; 5 Department of Neurology, Xi’an Jiaotong University First Affiliated Hospital, Xi’an, China; 6 Department of Psychiatry, Sir Run Run Shaw Hospital, Zhejiang University School of Medicine, Hangzhou, China; 7 Department of Neurology, Peking University First Hospital, Beijing, China; 8 Department of Neurology, Tianjin Medical University General Hospital, Tianjin, China; 9 School of Nursing, China Medical University, Shenyang, China; 10 Centre for Global Health, Usher Institute, University of Edinburgh, Edinburgh, UK; 11 Nossal Institute for Global Health, Melbourne School of Population and Global Health, University of Melbourne, Melbourne, Australia; 12 McGill Center for Studies in Aging, McGill University, Montreal, Canada

By the end of 2017, the population of China aged 65 and older has grown to 158 million, accounting for 11.4% of the total population [1]. Since age increases the risk of dementia, the increasing ageing population in China will contribute to a rise in the number of people with Alzheimer disease (AD) and other forms of dementia. The prevalence of dementia in China has thus increased considerably from the 1980s to 2010s [2].

Dementia care is a long journey challenged with significant economic burden, caregiver distress and increasing service demands [3-6]. These challenges will have a significant impact on the health care system in China, which aims to provide a continuum of services from screening and diagnosis of patients to longitudinal patient care and caregiver support. Strategically, the continuum of services for dementia has four major elements: risk assessment, timely diagnosis, post-diagnostic support, and caregiver support. Herein, we review the current needs, resources, and local practice related to the continuum of care in China.

## SITUATIONAL ANALYSIS

### Prevalence and incidence of dementia and related cognitive impairment in China

An early multi-site epidemiologic study focusing on people aged 55 years or older in 1997 demonstrated that China had a prevalence of AD (4.8%) and VaD (1.1%) that is comparable to Western countries [7]. Further analysis revealed that northern regions had a higher dementia prevalence than southern regions, especially for VaD, and that people with older age, female sex, or lower education level tended to have a higher prevalence of AD but not VaD [8]. After a follow-up of 4.5 years, the crude incidence was 7.8, 4.9, and 2.3 per 1000 person-years for dementia, AD, and VaD, respectively [9]. A multi-centre survey of residents aged 65 years or older conducted from 2008 to 2009 showed that the prevalence of dementia, AD, VaD, and MCI was 5.14%, 3.21%, 1.50%, and 20.8%, respectively [2,10]. The prevalence of dementia, MCI, and AD but not VaD was higher in rural areas than urban areas, and the education level might partly contribute to the difference.

There were also a host of single-site epidemiologic studies conducted in China in the past 30 to 40 years. According to systematic reviews and meta-analysis, the prevalence of dementia, AD, VaD, and MCI for the population aged 60 years and older in China was 2.8%-3.0%, 1.9%, 0.9%, and 12.7%, respectively, and the incidence was 9.87, 6.25 and 2.42 cases per 1000 person-years for dementia, AD, and VaD, respectively [11-14]. Moreover, the prevalence of AD and all forms of dementia increased with age (above 55 years old) and time (from 1990 to 2010), was higher in women than in men, and was comparable between urban and rural residents [11]. Chan et al. estimated that there were 9.19 million patients with dementia in China in 2010, including 5.69 million AD patients [11].

Wu et al. reported that the prevalence was approximately doubling every five-year age increase, and women had a higher prevalence than men above age 65. These results were based on a review and meta-analysis of 76 studies through 2012 [15]. They also observed that the prevalence of dementia was higher in the northern part of the country, lower in the southern region, and intermediate in the central area. Recently, it has been demonstrated that the age-stratified prevalence of dementia was higher in women than men and approximately doubling with every 5-year age increase. The pattern of decreasing prevalence of dementia from northern, central and southern China (unadjusted pooled estimate for northern China, 5.4%; central China, 3.8%; southern China, 3.7%) was reported in their updated review [16]. They also observed a high prevalence of dementia in western China (9.6%). Additionally, there was an increasing trend in the prevalence of dementia in China, even after adjusting for methodological factors and geographical areas (4.9% in 2010-2015 vs 2.8% before 1990). The current estimated prevalence of dementia is 5.3%, indicating that there are 9.5 million dementia patients in the population above age 60. The most recent estimate from the Institute for Health Metrics and Evaluation (IHME) of the University of Washington cites 10.427 million cases in China in 2015 [17].

**Figure:**
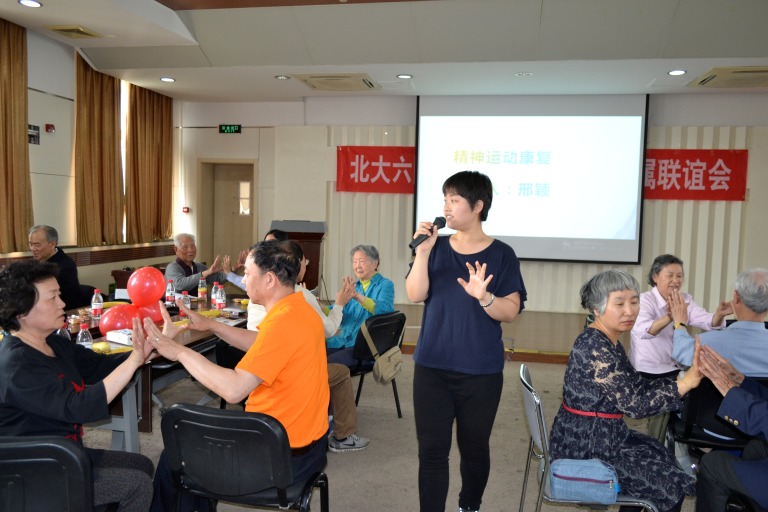
Photo: The volunteer provides training to the memory café participants on non-verbal communication with basic psychomotor therapy techniques (photo from the collection of the Dementia Care and Research Center, Peking University Institute of Mental Health, used with permission).

In general, the estimated prevalence and incidence of dementia were similar between the Chinese population and global population (prevalence, 3.9%; incidence, 7.5 per 1000 person-years at age 60 or older) [18]. Given improvements in education and prevention of vascular morbidity, the prevalence of dementia among the population 65 years or older declined during the past two decades. The prevalence decreased significantly in the United States (8.8% in 2012 vs 11.6% in 2000) and United Kingdom (6.5% in 2011 vs 8.3% expected in 2011 using data in 1991) [19-21]. However, the prevalence of dementia has increased in China along with the ageing population and increased life expectancy. The difference in prevalence rates between studies could also be partly attributed to the discrepancy in sample size, screening tools, methodologies, and diagnostic criteria. The total costs of patients with AD were estimated to be the US $167.74 billion in 2015 based on a nationwide investigation in China. This information indicated that the average cost per patient per year was approximately US $19 144.36 [3]. Taken together, China is facing a greater growing demand for the management of dementia than Western countries.

### The gap between diagnosis and treatment

The low rate of diagnosis is a significant concern in dementia care [22]. Chen et al. found that only 7% of Chinese older adults with dementia were diagnosed in a rural area [23]. On one hand, perspectives of persons with dementia (PWD), families, and physicians potentially impede the timely care seeking [24,25]. PWDs and their families consider cognitive decline as one of the phenomena of senility and normal ageing [26-28]. Shame and stigma may also be reasons for reluctance to seek care [29-34]. In China, approximately 76.6% of Chinese had a personal fear of Alzheimer disease [27]. Even in severe cases, families tend to use “senile confusion” [“*Lao Hutu*” (老糊涂) in Chinese pronunciation (characters)] rather than “dementia” to describe the clinical presentation.

Limited physician knowledge about dementia is another obstacle to timely diagnosis [25]. Few physicians in major hospitals in China know the diagnostic criteria of dementia [35]. Primary care physicians do not screen cognitive impairment routinely [36]. Thus, access to appropriate assessment and further diagnostic workup may be compromised.

Even in memory clinics, the delay in care seeking remains substantial. In a national registry study covering 28 well-established memory clinics, Zhao et al. observed that approximately two-thirds of persons with dementia delayed seeking care for more than 12 months, and one-quarter delayed seeking care for more than 36 months. The average duration from the first noticeable symptom to first visit seeking diagnosis or treatment was nearly 2 years [37].

### Care needs

At present, the care mode of affected older adults is divided into three categories: home care, community care, and institutional care.

Due to the influence of traditional Chinese culture, home care is the primary form of care, and most of the care is provided by family members (mostly spouses) and paid caregivers. People with mild and moderate dementia usually stay at home. Family members play a critical role in providing care.

In China, the typical community care, which uses community service resources to support daily life, typically interplays with home care [38]. In recent years, to effectively alleviate the growing demand for aged care, the government has complemented community day-care services. The day-care centre is an emerging form of aged care partly funded by social pension. Most services include catering and organizing leisure and entertainment activities for elderly individuals. Currently, only a limited number of day-care centres provide specific care for people with dementia.

Currently, there are several types of institutions designed for long-term care, including nursing homes, elderly apartments, old-age care communities, elderly care homes, and hospice care institutions. Regarding long-term care, only a few institutions have dementia care units. In most cases, persons living with dementia co-reside with those who are cognitively normal. However, managing challenging behaviours associated with moderate and severe dementia results in an emerging trend for increased demand for long-term care for dementia.

In China, most people with dementia receive informal care at home, while only those individuals with mild symptoms and those from high-income households have access to formal care at nursing homes. In a survey, only 2.0% of PWD who lived in a nursing home or hospital were cared for by professionals, and the remaining patients were cared for by non-professionals at home. Among those, 84.9% were cared for by family members; 8.3% lived alone; and 4.9% were cared for by hired nannies [39]. Spouses are the primary caregivers [40].

Unfortunately, due to a shortage of education programs and support services in China, most family caregivers and paid caregivers at home or nursing homes have limited knowledge and skills about care for someone with dementia. The burden for caregivers is severe, including great physical and mental pressure for caregivers [40,41].

Therefore, concerning the optimal care for PWD, there are significant challenges in implementing the continuum of care, including lack of trained care providers, insufficient training for the caregivers, limited support for the caregivers (especially for stress management and mental health), and insufficient investment for care funding.

## RESOURCES AND ADVANTAGES

### Ageing service policies

Recognizing the global social and economic burden, the World Health Organization has cited dementia as a public health priority [42,43]. Worldwide, more than 20 countries have developed a national dementia plan [44]. In China, dementia has always been considered a great social and health care challenge. Although there is no single national dementia plan, dementia has been addressed in several government documents [45].

The expansion of the new rural cooperative medical system (NRCMS), the urban employee basic medical insurance (UEBMI), and the urban resident basic medical insurance system (URBMI) has seen the achievement of universal health insurance in 2011 [46]. Having health insurance coverage is extremely important for older people, especially for those residing in rural areas and people who were never employed. These insurance schemes would provide platforms for to the development of culturally appropriate delivery systems for dementia care that are well integrated into the social medical service system.

In the 13^th^ Five-Year plan, a series of government guidance, covering a wide range of mental health and aged care services, was released and include dementia care as their integrative component (See a list of documents in **Table 1**).

**Table 1:** Primary government documents on mental health, ageing and aged care service released during 2013-2018

Governance body	Document title	Main content
The General Office of the State Council	National Mental Health Work Plan (2015-2020)	Focus on common mental disorders, such as dementia; pay attention to psychological and behavioural problems of elderly individuals; explore the appropriate prevention and treatment mode of common mental disorders
The General Office of the State Council	Guidance to promote the combination of medical care and pension services	Establish and improve the cooperation mechanism between medical institutions and pension institutions
The General Office of the State Council	Planning of national health service system (2015-2020)	Establish pension institutions, geriatric hospitals, geriatric nursing homes, rehabilitation institutions, etc.
Thirteen ministries	The “13^th^ Five-Year” Healthy Ageing Plan	Popularizing appropriate techniques for dementia and providing mental health and care services for elderly individuals
The General Office of NHFPC	Document to perform pilot work for old-age care	Implement targeted life care, family health care, mental comfort, emergency rescue and other activities for elderly individuals

NHFPC – National Health and Family Planning Commission (now as National Health Commission)

### Memory clinics

**Figure 1:**
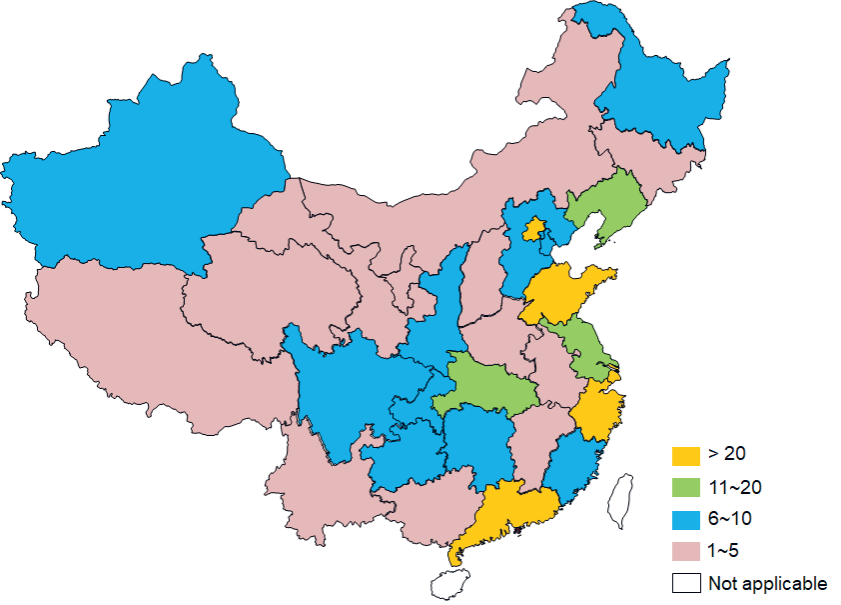
Geographical distribution of memory clinics in China. Colours code the number of memory clinics within the provincial administrative territory.

A memory clinic is the optimal setting for a comprehensive assessment and evaluation, creating a diagnosis and management plan, and following up in dementia care [47,48]. Since the first memory clinic was established in the 1990s, more than 260 memory clinics have been established in China to this point (see geographical distribution in the map, **Figure 1**).

Memory clinics in China provide a wide range of services by assessing all memory problems caused by brain injury, stroke, Parkinson disease, depression, mild cognitive impairment and dementia. Approximately half of the patients seen in current memory clinics have dementia [37].

Ideally, memory clinics provide a minimum service for PWD, including cognitive screening and assessment, neuroimaging examination, and medications. However, only one-third of physicians order cognitive tests for more than 75% of patients when making a diagnosis. Greater than three-quarters of physicians do not routinely evaluate the presence of neuropsychiatric symptoms. Therefore, the predicted increase in demand for memory clinic services means that further support, training, and supervision are required to guarantee the quality of service.

To improve support for patients living in rural communities in China, it is crucial to build a network of services that interact with more remote centres. Hence, a memory clinic network model has been proposed to improve accessibility to high-quality care for rural centres [49].

Ideally, multiple first-tier memory clinics (eg, in communities or less developed areas) will be established within the memory clinic network to provide essential care. These first-tier memory clinics will forge links with larger centres of excellence that already exist and have the skills and capabilities to screen, diagnose and provide an excellent level of care to their patients. Within the network, the newer clinics can access the facilities of these larger centres, including imaging resources and training. The centres of excellence should be linked into ‘circles of excellence' to share best practice and ensure consistency and efficiency to form a network that can share the increasing burden as patient numbers increase. Through these efforts, some community health centres have established a memory clinic, providing screening and long-term support for dementia.

### Social care resources

The current aged care system primarily relies on informal care from families and community-based social services, while the commercial caring system remains unaffordable to the most of China’s elderly population. This makes family and neighbourhood communities the optimal settings for delivering dementia care besides hospitals.

Underpinning the provision of informal care by families is the traditional ethics of filial piety and reciprocity between spouses [50]. In support of this effort, the National Aging Committee’s most recent guidelines has emphasised the urgent need to build age-friendly communities and systems for providing home-care services. To some extent, these strategies may also help engage society in community-based services for the PWD.

However, substantial efforts are needed to reach a high-level preparedness in the community. For example, there remains a debate about the Chinese word for *dementia* [28,51-53]. The word for dementia in Chinese is “*Chidai*,” which means “idiot, stupid.” Some people are concerned about its negative meaning and hope to change the word. Such intention reflects the vast misunderstanding of dementia and discrimination against patients with dementia in China. However, one can argue that the name “*chidai*” itself is not the origin of the stigma. It is the meaning endowed with the word that affects the perception of dementia. The impact of changing the name or empowering the word in China needs further exploration and studies.

Institutionalized care remains a last resort for most families of people with dementia. In addition to the nursing home established by the government, quite a few newly equipped nursing homes have been built in the past decade. These five-star nursing homes designed by international corporations drive the development of dementia units in nursing homes. Despite the modern infrastructure, the insufficiency of the well-trained staff remains a significant barrier. Therefore, capacity building is the primary challenge for improving care quality for PWD.

In recent years, long-term care issues have been recognised as a key concern. How to integrate medical and social care for elderly individuals has been a hot topic in this area. With the development of social worker organizations, different types of care have emerged. For example, in Beijing and Shanghai, small-sized day-care centers have recently been established in the community. These day-care centres take care of the PWD who live in the neighbourhood in the daytime. In some cities, such as Qingdao, some community-embedded care centres could provide respite care for elderly individuals when intensive care is temporarily needed.

## CONTINUUM OF CARE: STRATEGIES AND PRACTICE

In China, dementia care primarily depends on the health care providers, especially in the mild and moderate stage. Concerning the dementia care pathway, the World Alzheimer Report 2009 provides a seven-stage model for planning dementia services. Planning collaboratively with community-based health and social care and support services, Chinese health providers have modified the model into the continuum of care framework. Key areas include raising awareness, risk assessment, screening (community level), diagnosis (hospital level), post-diagnostic support and caregiver support (coordinated between hospital and community levels).

### Raising awareness

Signs and symptoms of dementia are commonly misunderstood as parts of normal ageing [28,52]. Lack of awareness and understanding of dementia and the stigma associated with it are significant barriers to early detection, appropriate treatment and management of dementia [54]. Increasing public awareness and understanding of dementia is the first step.

There are many examples of campaigns in China that aim to increase awareness and understanding of the condition. The nationwide campaign organized by Alzheimer Disease Chinese since 2002 is one such example. Every year, especially during World Alzheimer Month (September), numerous large-scale public lectures are delivered in communities; on TV, radio, and newspaper; and through social media. Pamphlets, booklets, and infographic cards are disseminated in the settings where elderly reside. The audience is estimated to be approximately 100 million or more. The message that dementia is a disease-causing disability and not an inevitable consequence of ageing is the main information of raising awareness. Currently, awareness-raising campaigns have been the primary driver for creating dementia-friendly communities.

### Risk assessment and screening

Alzheimer Disease Chinese proposed that a brief screening for cognitive decline should be integrated into the routine physical check-up [55]. A few community initiatives focus on the integrated care provided by general practitioners. Risk factors, such as smoking, drinking, cardiovascular diseases, and diet patterns, could be evaluated simultaneously with brief cognitive testing.

Although several instruments are useful for cognitive screening, AD8, clock drawing test, Mini-mental state examination (MMSE) and Montreal Cognitive Assessment (MoCA) are widely used [56]. For community health workers who have a heavy workload and limited time, AD8 and clock drawing tests are suggested. In the community-based dementia management toolkit, the WHO WPRO proposed a screening flowchart used in low- and middle-income countries to identify people who are at risk for dementia [57]. The toolkit has now been piloted in China.

As suggested in the toolkit, people at risk for dementia can be interviewed by community workers at community centres or home visits at the request of families. If the person consents to being screened, they will be asked the basic question of “Have you noticed a significant change in memory, behaviour or function in the last year?”. On the basis of the answer, the community worker will decide whether to refer individuals to a community doctor or specialist if available. Once an individual is suspected of having dementia, he or she will be referred to a specialist in the memory clinic.

### Diagnosis

In China, a formal diagnosis is made by specialists. Eligibility for and initiation of anti-dementia drug prescriptions is exclusively determined by specialists in the field, including neurologists, geriatric psychiatrists or geriatricians. As there is currently an absence of certification requirement for subspecialty training on dementia and cognitive impairment, it raises an issue of service capacity by health professionals, and quality of care may diverge across different areas in the country.

To harmonize the work-up of the diagnostic procedure, several practice guidelines on dementia management have been published (see **Table 2**). However, an efficient or effective method to monitor the physician's performance is lacking; it is difficult to estimate how the guidelines have been transferred into the routine practice. The poor implementation of dementia guidelines may cause additional barriers for accessing dementia care services in different areas nationwide.

**Table 2:** The list of dementia practice guidelines and consensus

Title	Publish date	Responsible organization
Dementia clinical practice and management guideline	2007	Chinese Society of Psychiatry
Management of dementia and cognitive disorders	2010	Committee of Dementia and Cognitive Disorders, Chinese Society of Neurology
		Alzheimer Disease Chinese (ADC)
Chinese expert consensus on memory check-up	2014	Alzheimer Disease Chinese (ADC)
Expert consensus on frontotemporal degeneration	2014	Working Group on Geriatric Neurology, Chinese Geriatrics Society
Chinese expert consensus on the diagnosis and treatment of Lewy body dementia	2015	Scientific Group on Neurodegenerative Diseases, Chinese Society of Micro-circulation
Expert consensus on care for people with cognitive disorders	2016	Committee of Cognitive Disorders, Chinese Society of Geriatrics
Guidelines on the application of brief cognitive testing in the diagnosis of dementia	2016	Guideline Working Group, Alzheimer Disease Chinese (ADC)
Chinese diagnostic guideline on vascular mild cognitive impairment	2016	Guideline Working Group, Alzheimer Disease Chinese (ADC)
Clinical expert consensus on the management of neuropsychiatric syndrome in neurocognitive disorders	2017	Psychogeriatric Interest Group, Chinese Society for Psychiatry
Expert consensus on the management of post-stroke cognitive disorders	2017	Scientific Group on Post-Stroke Cognitive Disorders, Chinese Stroke Society
Chinese Clinical Guideline on Dementia Management	2018	Alzheimer Disease Chinese (ADC)
Chinese guideline on dementia and cognitive disorders	2018	Committee of Dementia and Cognitive Disorders, Chinese Society of Neurology
		Alzheimer Disease Chinese (ADC)

**Figure 2:**
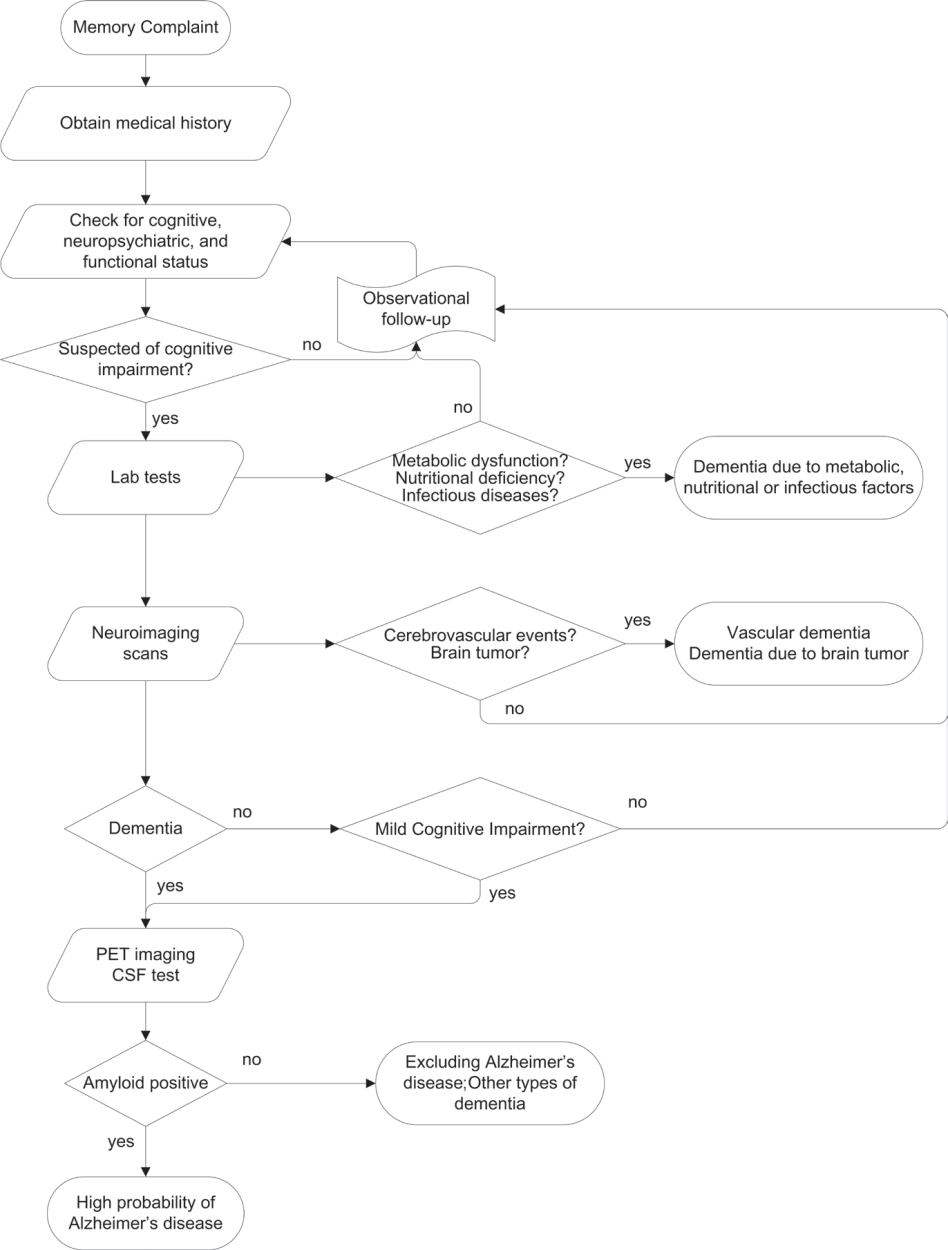
The workflow for making a diagnosis.

The diagnostic procedure work-up used in most memory clinics is illustrated in **Figure 2**. However, memory clinics need to function as a driver for better dementia care. Therefore, a group of Chinese and international experts who have abundant experience in managing memory clinics developed a consensus guide for operating the memory clinic [49]. The minimal and optimal requirements for the primary functions of a memory clinic are elaborated in full detail.

Although CSF and PET imaging may verify the biological diagnosis of Alzheimer disease, the application of these techniques are constrained due to several reasons, such as high cost and risk of physical discomfort. Only centres of excellence use these tests, and they are mainly for research purposes.

### Post-diagnostic support

In most memory clinics, once an individual is diagnosed with dementia, eligibility for anti-dementia drug prescriptions is determined by the specialist. Afterwards, a specialist in the memory clinic initiates the follow-up on pharmacological and non-pharmacological interventions, including cognitive training and psychosocial intervention [58,59]. Representative examples are shown in **Table 3**.

**Table 3:** Representative examples of the post-diagnostic support program (in alphabetic order based on city)

City	Hospital	Activity
Beijing	The Sixth Hospital of Beijing University	Caregivers support; Dementia café; Cognitive training
		Community outreach
Changsha	Changsha First Hospital	Caregiver support
Chongqing	The First Affiliated Hospital of Chongqing Medical University	Caregivers support
Hangzhou	Zhejiang Provincial Hospital	Caregiver education and support
Shanghai	Shanghai Mental Health Center	Family support with NGO; Nursing home service
Taiyuan	The First Hospital of Shanxi Medical University	Caregivers Education; WeChat consultation; Cognitive training
Tianjin	Tianjin Medical University General Hospital	Cognitive training and care support
Wenzhou	Wenzhou Medical University Affiliated First Hospital	Caregiver education and support
Xi’an	The First Affiliated Hospital of Xi’an Jiaotong University	Caregivers Education; WeChat consultation

For individuals experiencing psychological and behavioural problems, non-pharmacological interventions are the first-line option as explicitly specified by the consensus on the clinical management of neuropsychiatric symptoms [60]. However, there are minimal resources for psychosocial intervention for people living with dementia, even in long-term care settings. Therefore, a set of quality indicators for psychosocial intervention for people living with dementia are proposed to improve post-diagnostic support (**Table 4**) [61].

**Table 4:** Quality indicators of the psychosocial intervention for people living with dementia (adapted from Jiang et al [61])

Domain	Indicators
Assessment	A person with dementia and his/her primary caregiver should receive a comprehensive assessment, including background assessment, such as personal life experience, education and employment history, family and social support, interests, and hobbies (QI #1).
	Current situation evaluation, such as physical condition, functional status, medication, daytime activities, sleep and appetite, and nutritional status (QI #2).
	Regular evaluation of mental health statuses, such as anxiety and depression, preferably twice annually (QI #3).
	Assessment of primary caregiver’s mental health status and caregiving skills (QI #4).
Communication	The multidisciplinary intervention team should communicate with the family members and the person with dementia and convey knowledge of diagnosis and prognosis to them (QI #5).
Formulation and implementation of the intervention	The multidisciplinary team should formulate the psychosocial intervention with consultation with caregivers (caregivers in care institutions or at home) and the person with dementia (QI #6).
	The individualized plan should be based on the individual's interest and hobbies, cognitive function and physical conditions (QI #7).
	At least two types of psychosocial intervention should be provided to the person, including but not limited to daily living activities, recreational and social activities, and cognitive and behavioural training (QI #8).
	When implementing the psychosocial intervention, health and social care should be coordinated by designated staff, such as social workers, in the multidisciplinary team (QI #9).
	The designated staff should follow-up with the person with dementia and his/her primary caregiver (QI #10).
	The effectiveness of individualized psychosocial intervention should be regularly monitored, preferably once every six months (QI #11).
	Concerning the management of challenging behaviours, psychosocial intervention should always be the first choice and be documented (QI #12).
	The multidisciplinary team should regularly analyse the factors that contribute to, aggregate and improve the behaviours (QI #13).
	The intervention plan should be adjusted according to behaviour changes (QI #14).
	The team should implement the intervention according to behaviour changes (QI #15).
Caregiver support	Caregivers should be trained with caregiving skills (QI #16) and supported psychologically (QI #17).
Team management	The multidisciplinary team should receive continuous education on dementia care (QI #18).
	The team should be supervised on the practice of dementia care (QI #19).
	The team should obtain feedback from the person with dementia and primary caregivers, including the acceptance and satisfaction of the intervention plan (QI #20).

### Caregiver support

As the patient's disease severity progresses, the dependency on caregiver support will increase, and assistance may be required with activities of daily living, including administration of medication and financial and legal matters. Caring for a patient with AD impacts on the quality of life and health of the caregiver [40,41]. However, a range of interventions for caregivers, such as providing information about AD, advice about patient management, and the importance of self-care, can reduce caregiver burden [62].

In China, the tradition is for the family-based care for elderly individuals [50]. Often, a “nanny” is employed by the family to provide “one-to-one” care for patients with AD. However, it is likely that the nanny do not have any formal training in caring for AD patients. Many of these caregivers have a low level of education and literacy and may be isolated in rural communities. Therefore, caregiver support plays a vital role in China and is a crucial area where memory clinics could improve the standard of care.

Some memory clinics are already running monthly meetings for caregivers with a training or therapy program to prompt caregivers to consider how best they can look after the patient with AD at home. Caregiver support groups at the memory clinic or centre can be very helpful. Most groups are managed by a multi-disciplinary team, including a physician, trained nurses, and occasionally social workers.

The first memory café, namely, the dementia caregiver support group, was established in Beijing in 2000 (**Box 1**). For 18 years, the group has aimed to encourage caregivers to share their experience and concerns with others, educate caregivers with information about diagnosis and management and build a social support network for caregivers where their emotional distress could be relieved. Most groups meet regularly, ie, monthly or quarterly. As illustrated in **Table 5**, professionals give mini-lectures on the essentials of dementia, early signs of cognitive impairment and caregiving skills at group meetings. Volunteers then organize cognitive training and recreational activities for caregivers. Caregiver support groups have now been established in more than ten cities, including Beijing, Shanghai, Changsha, Taiyuan, Hangzhou, Wenzhou, Xi'an, and Tianjin. Recently, care support has become frequently provided via social media, such as WeChat groups.

Box 1 **Memory Café, Peking University Institute of Mental Health**

In 2000, Peking University Sixth Hospital established the first caregiver support group of dementia in China. It is a nonprofit support group composed of professionals and family members of patients to provide mutual assistance and support platform for families of dementia patients. For 18 years, professionals, patients, and their caregivers have gathered every Saturday morning on the second week of each month regardless of the weather. The activities of the caregiver support group are generally divided into two parts: mini-lectures and discussions on disease knowledge and care skills and cognitive and behavioural training, group psychological intervention or group activities, such as Finger exercises. Peking University Sixth Hospital Dementia Care and Research Center launched the child volunteer program in 2012. Every month child volunteers play music and art performance for the caregiver support group (see example in **Table 5**). By participating in various activities, the caregivers increase their knowledge of the disease, improve their care skills, release their emotions, decrease their pressure, and reduce the burden of care.

**Table 5:** Example of monthly meeting agenda of memory café

Date	Topic
April 8, 2017	1. How to reduce the caregiver burden?
	2. Relaxation training
	3. Children performance
May 13, 2017	1. Prevention of dementia
	2. Cognitive training
	3. Children performance
June 10, 2017	1. Effective communication with people living with dementia
	2. Reminiscence therapy
	3. Children performance

## FUTURE DIRECTIONS OF DEMENTIA CARE

In 2017, WHO adopted the Global Action Plan in the Public Health Response to Dementia 2017-2025 [43]. Improving access to care is the key to minimize the gap between diagnosis and treatment of dementia. In addition, as most older adults live in the community, making use of community resources might improve the pathway to care for dementia. Primary care physicians and non-specialists play a more significant role in diagnosing and managing dementia given the insufficient numbers of specialists. Furthermore, outreach in the community and regular home visits are considered essential for the timely identification of dementia.

Concerning community-based dementia care, representatives from multiple sectors of relevant stakeholders convened the High-level Roundtable Meeting on Dementia Action Plan in China in January 2018. The participants proposed five recommendations for implementing the dementia action plan: (1) It is imperative to integrate existing resources to promote dementia care; (2) Policy makers from different sectors will collaborate to promote the WHO dementia action plan and develop an implementation plan; (3) Building community-based care capacity is critical to increase access to care and service coverage; (4) Exploration of a novel collaborative management mechanism that aims to increase the efficiency of community dementia care is needed; and (5) Further exploration to utilize advanced technology is needed.

The WHO mhGAP program has used non-specialists to develop evidence-based guidelines for the management of dementia in order to scale up treatment and reduce the treatment gap [63,64]. The WHO Regional Office for the Western Pacific (WPRO) developed a “dementia toolkit for community workers in low- and middle-income countries” [57]. Therefore, implementing the mhGAP and dementia toolkit will be one of the major tasks in the coming decade.

In addition, the service of memory clinics should be made accessible for those who are suspected of dementia. More efforts are needed to implement the memory check-up plan in the community. Additionally, the Excellence Centers for Dementia Care are encouraged to function and liaison with the memory clinic networks to ultimately improve the diagnosis and standard care for dementia through education and training.

In addition, adequate support for nursing homes still needs to be leveraged. Social and medical care should be integrated into multidisciplinary teams to provide holistic care for persons with dementia.

Last but not the least essential, collaborative efforts of the relevant stakeholders should advocate for policy development, eg, developing national dementia plan, and implementation, eg, individual articles in the national mental health plan. Prioritizing dementia care on the policy development agenda will eventually allow the WHO Global Dementia Action Plan be effectively implemented in China. With the success of the high-level roundtable meeting in 2018, we can reasonably expect engagement from and partnership with the government sectors, health and social care facilities, academic institutions, non-governmental organizations, industry, and international institutions.
